# Individuals with psychosis receive less electric field strength during transcranial direct current stimulation compared to healthy controls

**DOI:** 10.1038/s41537-024-00529-2

**Published:** 2024-11-20

**Authors:** Rebecca Kazinka, Da Som Choi, Alexander Opitz, Kelvin O. Lim

**Affiliations:** 1 University of Minnesota, Department of Psychiatry & Behavioral Sciences, Minneapolis, MN USA; 2 University of Minnesota, Department of Biomedical Engineering, Minneapolis, MN USA

**Keywords:** Psychosis, Working memory, Neuroscience

## Abstract

Recent research has examined the effectiveness of transcranial direct current stimulation (tDCS) as an adjunctive treatment for antipsychotics, finding mixed results on cognitive, positive, and negative symptoms. We tested if individuals with psychosis have reduced electric field strength compared to healthy controls and assessed the potential causal factors. We hypothesized that either cortical thinning due to the disorder or increased scalp thickness due to secondary effects of the disorder were causal factors. Using the Psychosis Human Connectome Project dataset, we simulated electric field models for 136 individuals with psychosis, 73 first-degree relatives, and 43 healthy controls. We compared group differences of electric field strength at bilateral dorsolateral prefrontal cortex (dlPFC), targeted with two montages (Fp1-Fp2 & F3-Fp2) commonly used to treat cognitive impairment. We additionally compared groups on scalp, skull, and cerebrospinal fluid thickness at bilateral dlPFC and the three electrode locations. Mediation analyses assessed if tissue thickness and BMI were causal factors for group differences while controlling for age and sex. Individuals with psychosis had lower electric field strength for bilateral dlPFC for both montages. Scalp thickness was also greater for individuals with psychosis, but cerebrospinal fluid thickness was not significantly different. BMI was a significant mediator for the group difference seen in both scalp thickness and electric field strength. Future treatment studies using tDCS in the psychosis population should include electric field modeling to assess its effectiveness given the increased risk of obesity. Individualized montages based on head models may also improve effectiveness.

## Introduction

Individuals with psychosis often experience a disconnection from reality represented by delusions and hallucinations, but psychotic disorders can also be associated with cognitive impairment, in which antipsychotic medication is not always effective at improving functional outcomes^1,2^. In addition, antipsychotic medication can have unpleasant side effects that negatively affect treatment compliance^3^. Treatments using neuromodulation have been recently considered as an additional option, with the hope of reducing negative side effects. Several meta-analyses have shown efficacy of transcranial direct current stimulation (tDCS) in psychosis on cognitive symptoms, positive symptoms (i.e., delusions, hallucinations), and negative symptoms (e.g., anhedonia), although there were mixed but promising findings and uncertainty about specific montages^4–10^. This variability in the efficacy of tDCS may partly arise from physiological differences in psychosis.

Individual differences in anatomical features of the head and brain tissues have been shown to affect the electric field strength received from tDCS^11,12^. Importantly, different tissue types (skin, skull, gray matter, and white matter) conduct electricity to different degrees, meaning that natural changes in these tissue types can influence the flow of electric current in the brain^13,14^. Simulations of individualized electric field strength have predicted behavioral changes in motor-related tasks^15–17^, and some research has shown it can predict improvements when paired with working memory training^18,19^. Using this method, a recent study identified that individuals with psychosis have lower electric field strength in the bifrontal lobe, cerebellum, and dorsolateral and medial prefrontal cortex^20^. However, this study did not identify causal factors related to these group differences. Given that differences in electric field strength may affect the efficacy of tDCS, it is important to understand what factors influence these group differences to better predict treatment outcomes.

We propose two possible hypotheses that could affect outcomes. First, direct effects of the disorder on cortical thickness may affect electric field strength. Differences in brain morphology may determine the degree that gray matter and white matter are available to receive stimulation. Individuals with schizophrenia are associated with decreased gray matter volume in the medial temporal, superior temporal, and prefrontal regions^21–24^. Worsened cortical thinning is also associated with poorer outcomes, potentially reflecting that those individuals who would benefit most from additional treatment through tDCS would have a harder time receiving adequate stimulation^25,26^. Lastly, cortical thinning is associated with some antipsychotic medications^25^ and aging^27,28^. Alternatively, secondary effects of the psychotic disorder may contribute to changes in electric field strength. Approximately 40–60% of individuals with psychosis are considered overweight or obese^29,30^. Several factors, such as sedentary lifestyle, genetic susceptibility, and antipsychotic treatment^31,32^, are associated with weight gain in psychotic disorders^33^. Weight gain would in turn change scalp thickness, potentially affecting the ability for electrical stimulation to reach the gray matter tissue.

**Fig. 1:**
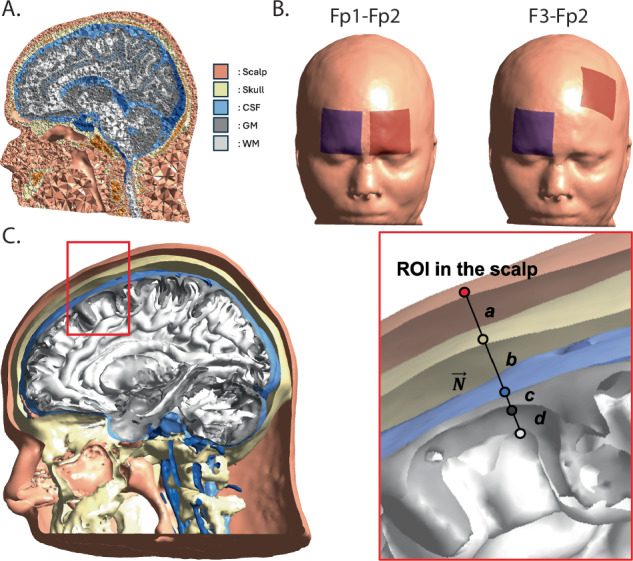
SimNIBS segmentation example and thickness calculation. **A** Example of head model showing tetrahedral elements assigned different tissue types (scalp, skull, CSF, gray matter, and white matter). **B** Locations of the electrodes for each montage. Red indicates the anode and blue indicates the cathode. **C** Calculation of tissue thickness in a head model. A normal vector $\vec{N}$, drawn perpendicular to the layers (tissues), is used to measure the thickness of each layer. For each layer, $\vec{N}$ is drawn perpendicular to that specific layer. The distances along these normal vectors represent the thickness of each layer: a) scalp, b) skull, c) CSF, and d) gray matter (GM).

The current study aimed to replicate findings of lower electric field strength in individuals with psychosis compared to healthy controls, with a particular focus on the dlPFC, a common target for tDCS studies on psychosis^7^ due to its role in executive processes related to cognitive functioning^34–36^. We analyzed structural MRI from the Psychosis-Human Connectome Project to ensure adequate power to assess small effects. We measured dlPFC electric field strength from two montages (Fp1-Fp2 & F3-Fp2) and tissue thicknesses (scalp, skull, cerebrospinal fluid, and cortex) at the dlPFC and electrode locations and tested the causal effect on group differences between controls and individuals with psychosis (Fig. 1). This sample also included first-degree relatives of the individuals with psychosis, who share genetics but have limited psychotic symptoms directly, allowing us to examine potential genetic factors. Broadly, we hypothesized that greater scalp-to-cortex thickness would lead to decreased electric field strength. More specifically, we had two potential hypotheses about the driver for this relationship. First, gray matter cortex would be thinner and cerebrospinal fluid (CSF) would be thicker, suggesting primary effects of the disorder on the brain caused these differences. Alternatively, if secondary factors related to psychotic disorders increased weight, we would find that scalp tissue would be thicker and impede the electrical stimulation. In turn, these changes in tissue thickness would mediate the group differences in electric field strength.

## Results

Individuals with psychosis had a higher body mass index (BMI) than healthy controls but did not differ on age or sex (Table 1). First-degree relatives were significantly older and had a higher BMI than healthy controls as well. Within individuals with psychosis, 91 reported taking antipsychotic medication, 22 reported not taking antipsychotic medications, and 23 did not disclose medication. Individuals taking antipsychotic medication (mean = 31.6, SD = 6.3) had a higher BMI than those who did not (mean = 28.0, SD = 7.0; W = 662, *p* = 0.014). Additional analyses controlled for age and sex.

**Table 1:** Abbreviated demographic information.

	Healthy Controls	Individuals with Psychosis	First-Degree Relatives	Healthy Controls vs. Psychosis	Healthy Controls vs. First-Degree Relatives
*N*	43	136	73	–	–
Mean age (SD)	38.2 (13.1)	38.8 (12.3)	44.6 (13.8)	W = 2867, *p* = 0.85	**W** = **1166,** ***p*** = **0.021**
Sex (Male %)	48%	56.6%	31.5%	X(1) = 0.515, *p* = 0.473	X(1) = 2.76, *p* = 0.097
Mean BMI (SD)	25.5 (5.5)	31.4 (7.4)	29.1 (6.6)	**W** = **1494,** ***p*** < **0.001**	**W** = **1032,** ***p*** = **0.002**

Bold type denotes significance.

### Individuals with psychosis have lower electric field strength at the dlPFC than healthy controls

**Fig. 2:**
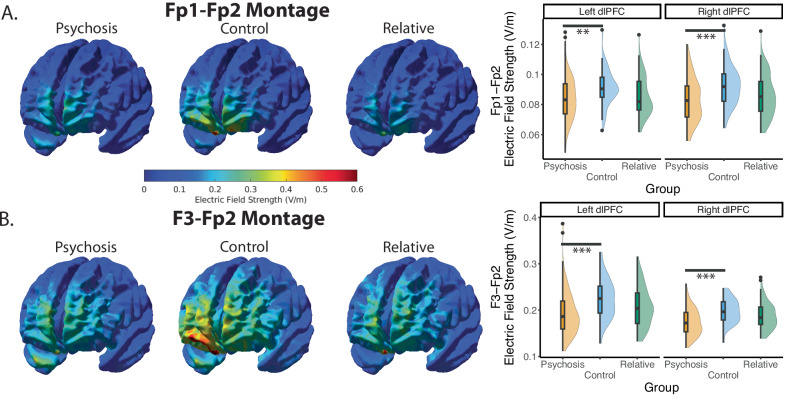
Comparisons of the electric field strength. **A** For the Fp1-Fp2 montage, individuals with psychosis had lower electric field strength at the left and right dlPFC compared to controls, but relatives did not when controlling for age and sex. **B** Results were similar when examining the F3-Fp2 montage, in which the individuals with psychosis had significantly lower electric field strength at bilateral dlPFC compared to healthy controls.

For the Fp1-Fp2 montage, the electric field strength at the left and right dlPFC was less in the individuals with psychosis (left: Estimate = −0.007, SE = 0.002, t = 3.13, *p* = 0.002, f^2^ = 0.050; right: Estimate = −0.009, SE = 0.002, t = 4.26, *p* < 0.001, f^2^ = 0.097; Fig. 2). Both left and right dlPFC electric field strength was lower in individuals with psychosis in the F3-Fp2 montage as well (left: Estimate = −0.031, SE = 0.008, t = 4.06, *p* < 0.001, f^2^ = 0.088; right: Estimate = −0.023, SE = 0.005, t = 4.93, *p* < 0.001, f^2^ = 0.132). There were no statistical differences between the first-degree relatives and the healthy controls at either of the dlPFC locations (*p*’s > 0.171, f^2^ < 0.011; Supplementary Table S6). When looking at whole brain results, individuals with psychosis had lower electric field strength compared to the healthy controls across the brain (Fig. 2).

**Fig. 3:**
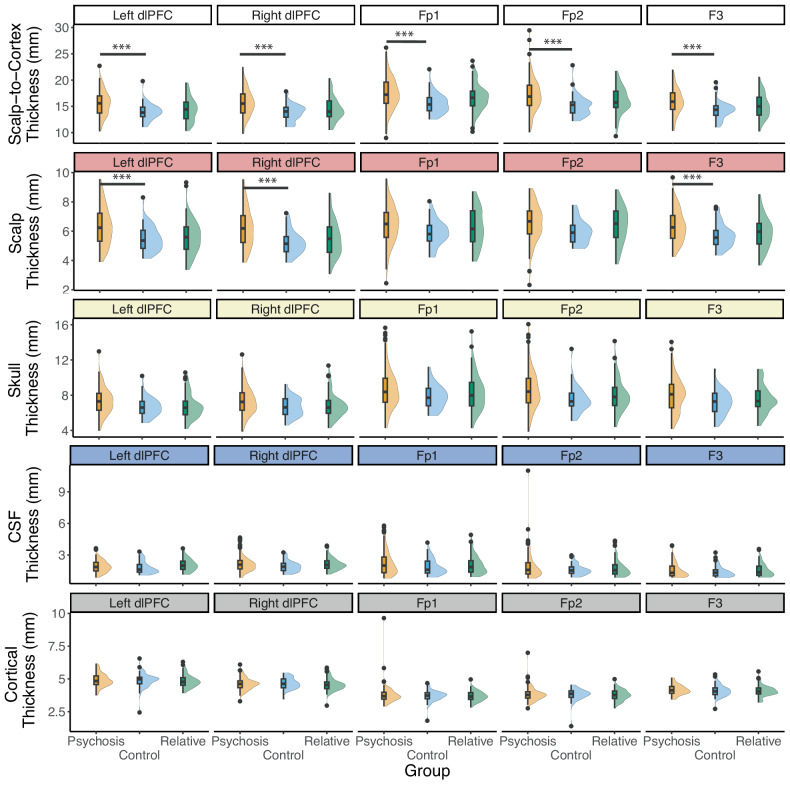
Tissue thicknesses. Individuals with psychosis had greater scalp-to-cortex thickness compared to healthy controls. First-degree relatives were not significantly different at any locations. When controlling for age and sex, scalp thicknesses were significantly different between the control and psychosis groups at the dlPFC and F3 electrode. There were no significant differences for the skull, CSF, or cortex thicknesses after Bonferroni correction.

The overall scalp-to-cortex thickness was significantly different at all ROIs (Left dlPFC: Estimate = 1.56, SE = 0.38, t = 4.11, *p* < 0.001, f^2^ = 0.090; Right dlPFC: Estimate = 1.78, SE = 0.400, t = 4.47, *p* < 0.001, f^2^ = 0.108; Fp1: Estimate = 1.73, SE = 0.461, t = 3.76, *p* < 0.001, f^2^ = 0.075; Fp2: Estimate = 1.91, SE = 0.485, t = 3.95, *p* < 0.001, f^2^ = 0.083; F3: Estimate = 1.62, SE = 0.395, t = 4.09, *p* < 0.001, f^2^ = 0.089; Fig. 3 top row). We further found that only differences in scalp at bilateral dlPFC and the F3 electrode survived Bonferroni corrections (Supplementary Table S1; Fig. 3). For first-degree relatives, no differences from the control group survived Bonferroni corrections.

### Scalp-to-cortex mediates electric field strength differences between individuals with psychosis and healthy controls

**Fig. 4:**
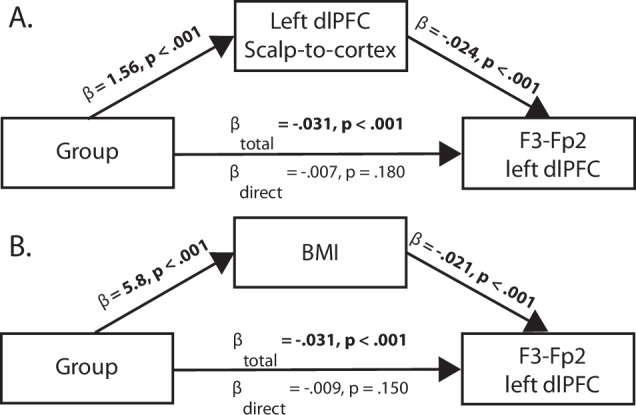
Example mediation results. **A** The left dlPFC scalp-to-cortex thickness showed that it was a full mediator for the group difference of the F3-Fp2 left dlPFC electric field strength. **B** Similarly, body mass index (BMI) was a significant mediator for the group difference of the F3-Fp2 left dlPFC electric field strength.

We first focused on the scalp-to-cortex thickness. We conducted a mediation analysis to examine the influence of the tissue thicknesses directly on estimated electric field strength to understand how these factors affected the delivery of stimulation (Table 2).We reported the effect of the mediator (average causal mediation effects; ACME), effect of group when including the mediator (average direct effects; ADE) and the effect of group without the mediator (total effects). We did not include the first-degree relatives in this analysis because there were no group differences in electric field strength. Scalp-to-cortex thickness at the bilateral dlPFC were full mediators for the group effect of the electric field strength for both montages (example in Fig. 4). The thickness at the respective electrodes similarly showed that scalp-to-cortex thickness was a full mediator for the group effect of the electric field strength. Thicknesses of the scalp at the dlPFC and electrodes were all full mediators for the group effect at the left dlPFC, and partial mediators for the group effect at the right dlPFC, for each montage (Supplementary Table S2). Skull thickness was less consistently a mediator of the group difference when adding Bonferroni correction, although it was mainly a partial mediator at the electrodes (Supplementary Table S3). In contrast, CSF was not a significant mediator for the electric field strength for any location (Supplementary Table S4).

**Table 2:** Mediation analyses of scalp-to-cortex thickness on electric field strength.

	Average Causal Mediation Effect	Average Direct Effect	Total Effect	Proportion Mediated
*Fp1-Fp2 Left dlPFC*				
Left dlPFC	**Est** = **−0.004,** ***p*** < **0.001**	Est = −0.003, *p* = 0.18	**Est** = **−0.007,** ***p*** = **0.002**	60.9%
Fp1 Electrode	**Est** = **−0.005,** ***p*** < **0.001**	Est = −0.002, *p* = 0.344	**Est** = **−0.007,** ***p*** = **0.002**	71.4%
Fp2 Electrode	**Est** = **−0.005,** ***p*** < **0.001**	Est = −0.002, *p* = 0.302	**Est** = **−0.007,** ***p*** = **0.002**	70.1%
BMI	**Est** = **−0.004,** ***p*** < **0.001**	Est = −0.003, *p* = 0.092	**Est** = **−0.007,** ***p*** = **0.002**	53.1%
*Fp1-Fp2 Right dlPFC*				
Right dlPFC	**Est** = **−0.005,** ***p*** < **0.001**	Est = −0.005, *p* = 0.016	**Est** = **−0.010,** ***p*** < **0.001**	44.9%
Fp1 Electrode	**Est** = **−0.005,** ***p*** < **0.001**	Est = −0.005, *p* = 0.022	**Est** = **−0.010,** ***p*** < **0.001**	52.0%
Fp2 Electrode	**Est** = **−0.005,** ***p*** < **0.001**	Est = −0.004, *p* = 0.018	**Est** = **−0.010,** ***p*** < **0.001**	55.5%
BMI	**Est** = **−0.003,** ***p*** < **0.001**	Est = −0.006, *p* = 0.012	**Est** = **−0.010,** ***p*** < **0.001**	38.6%
*F3-Fp2 Left dlPFC*				
Left dlPFC	**Est** = **−0.024,** ***p*** < **0.001**	Est = −0.007, *p* = 0.18	**Est** = **−0.031,** ***p*** < **0.001**	78.9%
F3 Electrode	**Est** = **−0.024,** ***p*** < **0.001**	Est = −0.007, *p* = 0.18	**Est** = **−0.031,** ***p*** < **0.001**	77.9%
Fp2 Electrode	**Est** = **−0.018,** ***p*** < **0.001**	Est = −0.012, *p* = 0.052	**Est** = **−0.031,** ***p*** < **0.001**	59.6%
BMI	**Est** = **−0.021,** ***p*** **<** 0.001	Est = −0.009, *p* = 0.15	**Est** = **−0.031,** ***p*** < **0.001**	69.5%
*F3-Fp2 Right dlPFC*				
Right dlPFC	**Est** = **−0.013,** ***p*** < **0.001**	Est = −0.010, *p* = 0.008	**Est** = **−0.023,** ***p*** < **0.001**	56.7%
F3 Electrode	**Est** = **−0.013,** ***p*** < **0.001**	Est = −0.010, *p* = 0.006	**Est** = **−0.023,** ***p*** < **0.001**	56.8%
Fp2 Electrode	**Est** = **−0.012,** ***p*** < **0.001**	**Est** = **−0.010,** ***p*** = **0.002**	**Est** = **−0.023,** ***p*** < **0.001**	54.7%
BMI	**Est** = **−0.012,** ***p*** < **0.001**	Est = −0.011, *p* = 0.010	**Est** = **−0.023,** ***p*** < **0.001**	53.5%

Average causal mediation effects (ACME) denote the mediation effect, average direct effects (ADE) is the effect of the group after controlling for the mediator, and total effects indicate the effects of group without the mediator. Bold type denotes analyses that survive Bonferroni correction.

Given that individuals with psychosis had greater BMI, we tested if this factor mediated the group effect on electric field strength (Table 2). BMI was a full mediator for the group effect on the electric field strength for all ROIs for both montages. Further mediation analysis showed that BMI was also a full mediator for the scalp-to-cortex thickness group differences at the left dlPFC (ACME = 1.06, p < 0.001; ADE = 0.505, p = 0.100; proportion mediated = 67.6%), F3 (ACME = 1.05, p < 0.001; ADE = 0.570, p = 0.072; proportion mediated = 64.7%), Fp1 (ACME = 0.914, p < 0.001; ADE = 0.820, p = 0.026; proportion mediated = 52.7%) and Fp2 electrodes (ACME = 0.959, p < 0.001; ADE = 0.954, p = 0.014; proportion mediated = 50.1%). BMI was a partial mediator for the scalp-to-cortex group effect at the right dlPFC (ACME = 1.01, *p* < 0.001; ADE = 0.775, *p* < 0.001; proportion mediated = 56.5%). Finally, BMI was a significant mediator for the group effect on the scalp at all locations and skull thicknesses at bilateral dlPFC and the F3 electrode (Supplementary Table S5).

## Discussion

This study examined differences in the electric field strength between individuals with psychosis, their first-degree relatives, and healthy controls, and it assessed potentially causal factors for these differences. Using the large sample from P-HCP, we simulated electric field models to show that individuals with psychosis have a significantly lower electric field strength compared to healthy controls, but this difference was not found between their first-degree relatives and healthy controls. Further assessment of the individuals with psychosis found that individuals with psychosis also had higher BMI, particularly for those who were prescribed antipsychotics. Mediation analyses showed that BMI was a significant mediator for group differences in electric field strength and scalp-to-cortex thickness, which was itself a significant mediator for electric field strength group differences. When we assessed the individual tissues, we found that individuals with psychosis had thicker scalps at bilateral dlPFC and the F3 electrode, and these thicknesses were mediators for the group difference in electric field strength, but there were no consistent differences in skull, CSF, or cortical thicknesses when including Bonferroni corrections. BMI was also a mediator for all group differences in scalp thicknesses. Overall, these results suggest that BMI and its effect on tissue thickness is a driving factor in the group differences in electric field strength; our findings do not support the hypothesis that cortical thinning affected stimulation.

As hypothesized, the largest effect sizes were seen in the scalp thicknesses and were strongly associated with BMI, which is supported by previous studies^37,38^. However, the hypothesized role of CSF or cortical thinning was not supported. Therefore, it is more likely that secondary factors of psychotic disorders increase BMI and in turn affect the resultant electric field strength. We found that those with a chlorpromazine equivalence greater than zero had greater BMI, suggesting antipsychotics contributed to this effect. Atypical antipsychotics like olanzapine and clozapine are more likely to cause weight gain as a side effect^39,40^, but we are not able to explicitly test which medications were most influential on this group difference in BMI due to the secondary nature of our analysis of the P-HCP dataset. However, medication may not be the only driving cause of increased BMI, as drug-naive individuals with first-episode psychosis also have a higher odds of metabolic syndrome, which may contribute to increased BMI^41,42^. While medication may be an important factor in the observed difference, other contributing factors include genetic factors, socioeconomic risk factors, sedentary lifestyle and increased food intake^43–45^. Of note, first-degree relatives also had a higher BMI than the control group, consistent with findings that genetic factors contribute to differences in metabolism in unaffected first-degree relatives^46^. We did not find the same differences in electric field strength, however, suggesting that there may be contributions of genetic factors as well as environmental factors that led to greater differences in the psychosis group specifically.

As the field aims to implement tDCS or other neuromodulation techniques to treat cognitive impairment in psychosis, it is important to consider the effects of BMI and scalp-to-cortex thickness on the resultant effects of tDCS. Even if it is not directly related to symptomatology, our results show a systematic effect that could limit the efficacy of tDCS as a treatment because the effects of tDCS (electric field changes) are not reaching the intended brain targets at a sufficient strength. These findings may suggest that stimulation parameters should be individualized to combat individual differences in tissue thickness. Past studies have suggested individualizing treatment to optimize current amplitude and montage based on electric field modeling^47^. For example, Joshi and colleagues^48^ administered reverse-calculated dosages to equal the group average from 1 mA compared to fixed 1 mA stimulation, and found that there was reduced variability in the modeled electric field strength, but that this effect did not translate to changes in behavioral outcomes. Other studies have measured reverse-calculated dosages and found they related to behavior, but the stimulation amplitude was theoretical, often calculated by normalizing to the group average^15^. A downside to this approach is that it would increase the administered current, which is often aimed at 1–2 mA, but is considered safe up to 4 mA^49^. Increased current may also increase side effects of tDCS, such as skin irritation and headaches, but is suggested to still be tolerable at 4 mA^50^. In contrast, research using individualized montages have shown that different participants respond better to different montages, and by using the most effective montage at a single session it led to greater improvement in behavior^51,52^. We found that the F3-Fp2 montage had higher electric field strength than the Fp1-Fp2, which may more accurately target the left dlPFC and could potentially have a stronger effect, although the effect size of the group difference in scalp thickness was higher at the F3 electrode than Fp1 or Fp2 electrodes. In order to optimize stimulation strength, both individualizing the montage and, to a lesser degree, modulating the current amplitudes may be additional options to improve treatment outcomes.

An important caveat of this work is that we did not test if these group differences led to specific changes in cognitive functioning. However, recent research has shown that when using frontotemporal tDCS in individuals with schizophrenia to treat auditory hallucinations, treatment responders had higher modeled electric field strength in the left transverse temporal gyrus at baseline^53^, suggesting that modeled electric field strength is associated with outcomes in this population. The authors hypothesized that this difference was caused by cortical thinning, but they did not measure tissue thickness to assess its influence. In our study, we only targeted the prefrontal cortex, and therefore we cannot generalize our findings to other regions of the brain. Cortical thinning in psychosis is greater in frontotemporal regions of the brain, and therefore might have a greater impact on outcomes in the temporal lobe^54^. In addition, scalp and skull thickness varies across the head and may have different impacts depending on the location^55^. Due to these factors, we encourage future studies to integrate finite element modeling when planning neuromodulation studies, including individualizing treatment and analyses when available. Future research would benefit from using finite element modeling to assess the influence of individual differences in electric field strength on behavioral outcomes, as this may explain apparent mixed findings in the literature regarding neuromodulation as a treatment for symptoms related to psychosis^56^. Future work could also integrate these findings to assess if biomarkers (such as BMI or body fat percentage) may provide clinical utility to reduce the burden of expensive imaging such as MRIs.

Additional limitations include the use of many statistical tests, as this was an exploratory study. While a larger sample size can increase power and reduce the rate of type II error (false negatives), increasing the number of statistical tests can lead to an increase in the rate of type I error (false positives), and thus we implemented Bonferroni corrections for multiple comparisons to strengthen our findings. Our findings corroborate one another, particularly that group differences in electric field strength and scalp-to-cortex thicknesses were seen across all ROIs and montages. In addition, it is possible that there are changes in conductivity within the brain in individuals with psychosis, which we did not model^13^. SimNIBS also does not include fat as a separate tissue type, which may contribute to slight changes due to elevated BMI, although evidence suggests this is a small effect^57^. Lastly, we only modeled tDCS using relatively broad saline electrodes, but other types of neuromodulation exist, such as transcranial magnetic stimulation and high definition (HD) tDCS. HD-tDCS over the dlPFC has been shown to be effective in treating cognitive deficits in schizophrenia^58^, and may be another consideration for treatment that we did not assess. Transcranial magnetic stimulation functions somewhat differently than tDCS yet is also affected by scalp-to-cortex thickness, and therefore may require similar considerations^59^. We only focused on targeting the left dlPFC with the anode, as it has previously been associated with successful improvements in cognition, but other symptom targets may be focused on other brain regions.

In conclusion, our study found that there is a small reduction in electric field strength in individuals with psychosis compared to healthy controls, consistent with previous research^20^. We further showed that BMI is a main contributing factor in this difference, which in turn leads to greater scalp-to-cortex thickness (and most strongly scalp), limiting the conductivity of the electric current in tDCS. Providers and researchers using tDCS with the psychosis population should take care to consider BMI as a potential factor limiting the effectiveness of tDCS. Of note, there was not sufficient evidence that specific factors related to psychosis led to the reduced electric field strength, but rather secondary effects potentially due to medication and changes in daily functioning. Future studies using tDCS in psychiatric populations would benefit from including finite element modeling to confirm that these results apply to real-world outcomes. Future research may also consider potential clinical measures that could aid in individualizing treatments without expensive imaging such as MRIs. These changes may improve the effectiveness of tDCS on cognitive deficits in psychosis, which has been shown to be effective in psychiatric populations^7,60^. With small adjustments, individuals with psychosis may have a greater chance of benefiting from this treatment as well.

## Methods

### Participants

Participants were selected from the Psychosis-Human Connectome Project (P-HCP), a publicly available database that includes individuals with psychosis and their first-degree relatives, along with healthy controls^61^. Individuals with psychosis were recruited if they had a diagnosis of schizophrenia, schizoaffective disorder, or bipolar I disorder with a history of psychotic symptomatology. First-degree relatives were biological siblings, parents, or offspring of someone with psychosis, and were recruited regardless of psychopathology. Healthy controls were individuals with no history of an illness with psychotic features or major depressive disorder, as well as no first-degree biological relatives with a history of psychiatric hospitalization for a psychotic or affective disorder. Individuals also did not meet exclusion criteria related to MR safety, neurological conditions, substance dependence or other disabilities affecting participation (see Demro et al.^61^ for details).

We included all participants who reported a body mass index (BMI) and had a structural MRI, leading to 43 healthy controls, 136 individuals with psychosis, and 73 first-degree relatives (Table 1). Individuals with psychosis included schizophrenia (83), schizoaffective disorder (17), bipolar I disorder with psychosis (35), and schizophreniform with depression not otherwise specified (1). First-degree relatives were diagnosed with schizophrenia (1), bipolar I disorder without psychosis (1), major depressive disorder (25), dysthymic disorder (2), adjustment disorder (1), anxiety disorders (2), social phobia (1), and eating disorder not otherwise specified (1); all other first-degree relatives denied current psychiatric illness (39). Healthy controls reported generalized anxiety disorder (1); all other healthy controls denied current psychiatric illness (42).

A sensitivity analysis suggested that our main analyses (linear regressions) were sensitive to effect sizes as low as f^2^ = 0.04 between the healthy controls and individuals with psychosis with power = 0.80 and alpha = 0.05. For the comparison between healthy controls and first-degree relatives, our analyses are sensitive to effect sizes as low as f^2^ = 0.07.

### Procedure

Participants did not interact with our experimental team and were not stimulated directly. As a part of P-HCP, participants were scanned on a Siemens 3 Tesla Prisma MR scanner using a 32-channel head-coil at the Center for Magnetic Resonance Research at the University of Minnesota. We utilized the structural scans, including the whole-brain anatomical multi-echo T1_w_ (MPRAGE; TE = 1.18/3.6/5.39/7.18 ms, TR = 2500 ms, flip angle = 8°, voxel size = 0.8 mm isotropic, scan time = 8:22 min) and T2-weighted turbo spin-echo with volumetric navigators (SPACE; TE = 564 ms, TR = 3200 ms, variable flip angle, voxel size = 0.8 mm isotropic, scan time = 6:35 min) scans acquired using the HCP Lifespan protocol^61^.

The electric field strength across the brain was estimated using SimNIBS 4.0.0^62^ integrated with Matlab (v. 2022). T1-weighted and T2-weighted structural scans were used in SimNIBS’ *charm* pipeline, which segments the head into different tissues and creates a tetrahedral finite element method (FEM) head model. Segmentations are labeled as skin, compact bone, spongy bone, cerebrospinal fluid, blood, eyes, gray matter, and white matter (Fig. 1A). SimNIBS solves electric field problems by using the finite element method (FEM), a computational method for approximating solutions to problems with boundary conditions in engineering. By solving a quasi-static Laplace equation given by **E** = −∇φ with Dirichlet boundary conditions at the electrodes (**E** represents the electric field, and φ denotes the electric potential), the electric fields in the entire domain can be calculated^63,64^. Our analysis focused on the magnitude of the tDCS electric field.

The montage of electrodes, including current intensity, position, and shape of electrodes, was determined based on a literature review of common montages targeting the dlPFC in individuals with psychosis^5,7,8^. For position, we used the 10-10 system to place electrodes for two montages: Fp1-Fp2 and F3-Fp2. While Fp1-Fp2 is further from the dlPFC than the F3-Fp2 montage, it has the advantage that it is easier to place the electrodes and does not have the added resistivity of hair^65^. Some studies have used Fp1/Fp2 as stimulation locations with a reference on the upper arm and found small to medium effects on cognition^66,67^. SimNIBS was then used to simulate the electric field strength for each subject using 25 cm^2^ (5 × 5 cm) 5-mm-thick sponge electrodes with a current = 2 mA and the default values of conductivities of tissues and electrodes^12,68,69^ (Fig. 1B). In both montages, the anode was over the left side, while the cathode was over the right side, because past studies have found greater efficacy at targeting the left dlPFC with the anode in tDCS studies on schizophrenia^5,7,8,70^. We extracted the electric field strength at the bilateral dlPFC by averaging across a 10 mm radius centered at previously reported MNI coordinates (left: [−44, 27, 33]; right [28, 31, 46]; ^71^) using SimNIBS’ mni2subject_coords. We additionally tested group differences at the bilateral dlPFC using a 20 mm radius and found that the group difference and mediation results were consistent (Supplementary Tables S6 and S7). Although the left dlPFC is often the target in tDCS studies, we examined bilateral dlPFC to compare laterality between the montages, especially because the F3-Fp2 montage is asymmetrical, while the Fp1-Fp2 montage is not.

To assess the influence of the anatomical components affecting the electric field strength, we calculated the thicknesses of the scalp, skull, CSF, and gray matter cortex; we also calculated an aggregate measure of scalp-to-cortex thickness (Fig. 1C). The thicknesses of the scalp, skull, CSF, and gray matter cortex were calculated based on specific coordinates of interest, such as those corresponding to electrode placements or other user-defined coordinates. We defined a region of interest (ROI) on the scalp with a 10 mm radius centered on the bilateral dlPFC coordinates and three electrodes (Fp1, Fp2, and F3). Scalp thickness was defined as the average of the minimum distances from the center of each scalp element within the ROI to the closest skull surface tetrahedral element. To ensure that these distances were perpendicular to the scalp surface, the orientation of the normal vectors at the scalp elements was verified to be directed toward the corresponding points on the skull element. Similarly, the thickness of the skull and CSF were determined by calculating the average of the minimum distances from the center of each element, as selected in the prior step, to the closest element of the neighboring tissue type. Lastly, the scalp-to-cortex thickness was subsequently calculated by summing the thicknesses of the scalp, skull, and CSF.

### Statistical analysis

#### Group comparisons

We first investigated if there were significant group differences in electric field strength at the dlPFC for each montage using a linear regression with a group variable (control = 0, patient = 1) and controlled for age and sex. Next, we sought out what other potential variables were different between groups that might influence this relationship. To compare the controls and individuals with psychosis, we compared demographic characteristics using a Wilcoxon rank sum test, as the data were not normally distributed (one-sample Kolmogorov–Smirnov; age: D = 1, *p* < 0.001; BMI: D = 1, *p* < 0.001) and a chi-squared test of independence for gender. We also assessed differences in BMI based on the reported use of antipsychotic medication (chlorpromazine equivalence > 0) for the individuals with psychosis only^72,73^. In addition, we assessed scalp-to-cortex thicknesses at the dlPFC and electrode placements using a linear regression with a group variable and controlled for age and sex. We included controls for age and sex because these factors affect features such as cortical thickness^74–76^, skull thickness^77–79^, CSF thickness^80^, and scalp thickness^81,82^, all of which may ultimately influence neuromodulation^83^. Individual tissue thicknesses are reported in the supplement (Supplementary Table S1).

We repeated these analyses but compared the first-degree relatives to healthy controls. Our aim for this comparison was to examine the influence of genetic factors that may contribute to these differences, rather than treatment-related factors. All analyses were corrected using Bonferroni correction for each type of analysis, such that alpha = 0.003 (0.05/16).

#### Mediation analysis

Given that there were group differences, we next conducted a mediation analysis to understand how these significant factors influenced the resultant electric field strength. We examined the mediation effects of variables that were significantly different between the healthy controls and individuals with psychosis when predicting each of the four electric field strength outcomes (left and right dlPFC for the two montages). We used the mediation package (Tingley et al.^84^) in R (v. 4.1.0) to calculate the average causal mediation effects (ACME), average direct effects (ADE) and total effects, which describe the influence of the mediator, effects of group when including the mediator, and the effects of group when not including the mediator, respectively. We controlled for age and sex for all analyses. We implemented a nonparametric bootstrapping for variance estimation with 1000 simulations. All analyses were also assessed using Bonferroni correction for each analysis, such that alpha = 0.004 (0.05/12).

## Supplementary information

Supplemental Materials

## Data Availability

The Psychosis – Human Connectome Project is available at humanconnectome.org. The data will be made available at the NIH Data Archive.
