# Supplementary material for: Individuals with psychosis receive less electric field strength during transcranial direct current stimulation compared to healthy controls
Source: Schizophrenia (Heidelb). 2024 Nov 20;10(1):111. doi: 10.1038/s41537-024-00529-2 (PMC11579372; doi:10.1038/s41537-024-00529-2)
Supplement: Supplementary file 1 — Supplemental Materials [file 41537_2024_529_MOESM1_ESM.pdf]

Supplemental Materials for: Individuals with psychosis receive less electric field strength during transcranial direct current stimulation compared to healthy controls

Rebecca Kazinka[1,2], Da Som Choi[2], Alexander Opitz[2], & Kelvin O. Lim[1]

1. University of Minnesota, Department of Psychiatry & Behavioral Sciences
2. University of Minnesota, Department of Biomedical Engineering

Supplementary Materials

Table S1. Tissue thicknesses by group.

	Healthy Controls	Individuals with Psychosis	First-Degree Relative	Healthy Controls vs. Psychosis	Healthy Controls vs. First-Degree Relatives
			Left dlPFC		
Scalp	5.48 (.86)	6.32 (1.31)	6.60 (1.17)	**Estimate = .803,<br>SE = .210,<br>t = 3.82,<br>p < .001,<br>f^2 = .077**	Estimate = .181,<br>SE = .213,<br>t = .852,<br>p = .396,<br>f^2 = -.002
Skull	6.6 (1.10)	7.33 (1.50)	6.76 (1.43)	Estimate = .674,<br>SE = .242,<br>t = 2.79,<br>p = .006,<br>f^2 = .038	Estimate = -.050,<br>SE = .253,<br>t = .196,<br>p = .845,<br>f^2 = -.008
CSF	1.82 (.59)	1.92 (.57)	2.06 (.59)	Estimate = .085,<br>SE = .097,<br>t = .876,<br>p = .382,<br>f^2 = -.001	Estimate = .128,<br>SE = .106,<br>t = 1.21,<br>p = .23,<br>f^2 = .004
Cortex	4.89 (.62)	4.90 (.50)	4.84 (.49)	Estimate = .003,<br>SE = .087,<br>t = .037,<br>p = .971,<br>f^2 = -.006	Estimate = .083,<br>SE = .100,<br>t = .827,<br>p = .410,<br>f^2 = -.003
			Right dlPFC		
Scalp	5.21 (.80)	6.21 (1.33)	5.48 (1.19)	**Estimate = .944,<br>SE = .208,<br>t = 4.54,<br>p < .001,<br>f^2 = .121**	Estimate = .362,<br>SE = .209,<br>t = 1.73,<br>p = .086,<br>f^2 = .018
Skull	6.69 (1.06)	7.31 (1.55)	6.82 (1.43)	Estimate = .617,<br>SE = .245,<br>t = 2.52,<br>p = .013,<br>f^2 = .030	Estimate = -.055,<br>SE = .254,<br>t = .218,<br>p = .827,<br>f^2 = -.008
CSF	1.92 (.52)	2.14 (.74)	2.17 (.59)	Estimate = .220,<br>SE = .119,<br>t = 1.84,	Estimate = .126,<br>SE = .105,<br>t = 1.20,

				p = .067, f^2 = .014	p = .234, f^2 = .004
Cortex	4.67 (.43)	4.63 (.46)	4.57 (.49)	Estimate = -.042, SE = .073, t = -.567, p = .572, f^2 = -.004	Estimate = .042, SE = .082, t = .511, p = .61, f^2 = -.006
			Fp1 Electrode		
Scalp	5.89 (.90)	6.45 (1.30)	6.26 (1.29)	Estimate = .540, SE = .213, t = 2.54, p = .012, f^2 = .031	Estimate = .241, SE = .228, t = 1.06, p = .292, f^2 = .001
Skull	7.78 (1.33)	8.72 (2.28)	8.33 (2.08)	Estimate = .830, SE = .347, t = 2.39, p = .018, f^2 = .027	Estimate = .744, SE = .332, t = 2.24, p = .027, f^2 = .036
CSF	1.88 (.77)	2.25 (1.15)	2.07 (.88)	Estimate = .365, SE = .178, t = 2.05, p = .042, f^2 = .018	Estimate = .018, SE = .144, t = .129, p = .898, f^2 = -.009
Cortex	3.69 (.46)	3.78 (.66)	3.69 (.39)	Estimate = .083, SE = .110, t = .756, p = .451, f^2 = .003	Estimate = .037, SE = .083, t = .451, p = .653, f^2 = -.007
			Fp2 Electrode		
Scalp	5.96 (.82)	6.57 (1.26)	6.41 (1.25)	Estimate = .604, SE = 204, t = 2.96, p = .003, f^2 = .044	Estimate = .399, SE = .221, t = 1.80, p = .074, f^2 = .020
Skull	7.50 (1.50)	8.63 (2.21)	7.99 (1.86)	Estimate = 1.01, SE = .339, t = 2.99, p = .003, f^2 = .045	Estimate = .579, SE = .322, t = 1.80, p = .074, f^2 = .020
CSF	1.64 (.54)	1.94 (1.21)	1.77 (.82)	Estimate = .296, SE = .183, t = 1.62, p = .107,	Estimate = .04, SE = .126, t = .327, p = .744,

				f^2 = .009	f^2 = -.008
Cortex	3.79 (.50)	3.83 (.51)	3.77 (.42)	Estimate = .031,<br>SE = .089,<br>t = .347,<br>p = .729,<br>f^2 = .005	Estimate = .065,<br>SE = .086,<br>t = .752,<br>p = .454,<br>f^2 = -.004
			F3 Electrode		
Scalp	5.69 (.81)	6.34 (1.08)	5.89 (1.07)	**Estimate = .616,<br>SE = .175,<br>t = 3.51,<br>p < .001,<br>f^2 = .064**	Estimate = .250,<br>SE = .197,<br>t = 1.27,<br>p = .208,<br>f^2 = .005
Skull	7.13 (1.37)	8.03 (1.94)	7.59 (1.59)	Estimate = .915,<br>SE = .314,<br>t = 2.92,<br>p = .004,<br>f^2 = .043	Estimate = .209,<br>SE = .290,<br>t = .720,<br>p = .473,<br>f^2 = -.004
CSF	1.44 (.55)	1.54 (.66)	1.57 (.65)	Estimate = .086,<br>SE = .106,<br>t = .817,<br>p = .415,<br>f^2 = -.002	Estimate = -.021,<br>SE = .104,<br>t = .199,<br>p = .842,<br>f^2 = -.008
Cortex	4.11 (.48)	4.16 (.38)	4.08 (.43)	Estimate = .050,<br>SE = .067,<br>t = .752,<br>p = .453,<br>f^2 = -.003	Estimate = .077,<br>SE = .084,<br>t = .926,<br>p = .357,<br>f^2 = -.001

Note. Values are means and standard deviations. Bold text denotes analyses that survive Bonferroni correction.

Table S2. Scalp thickness mediations.

	Average Causal Mediation Effect	Average Direct Effect	Total Effect	Proportion Mediated
		Fp1-Fp2 Left dlPFC		
Left dlPFC	**Est = -.002, p = .002**	Est = -.005, p = .030	**Est = -.007, p < .001**	28.9%
Fp1 Electrode	**Est = -.002, p = .002**	Est = -.005, p = .020	**Est = -.007, p = .002**	27.6%
Fp2 Electrode	**Est = -.003, p < .001**	Est = -.005, p = .030	**Est = -.007, p < .001**	35.6%
		Fp1-Fp2 Right dlPFC		
Right dlPFC	**Est = -.003, p < .001**	**Est = -.007, p = .002**	**Est = -.010, p < .001**	30.8%
Fp1 Electrode	Est = -.002, p = .016	**Est = -.008, p < .001**	**Est = -.010, p < .001**	17.8%
Fp2 Electrode	**Est = -.002, p < .001**	**Est = -.008, p < .001**	**Est = -.010, p < .001**	23.9%
		F3-Fp2 Left dlPFC		
Left dlPFC	**Est = -.016, p < .001**	Est = -.016, p = .036	**Est = -.031, p < .001**	50.2%
F3 Electrode	**Est = -.015, p < .001**	Est = -.016, p = .016	**Est = -.031, p < .001**	48.4%
Fp2 Electrode	**Est = -.009, p = .004**	Est = -.022, p < .001	**Est = -.031, p < .001**	29.6%
		F3-Fp2 Right dlPFC		
Right dlPFC	**Est = -.010, p < .001**	**Est = -.013, p = .002**	**Est = -.023, p < .001**	41.6%
F3 Electrode	**Est = -.009, p = .004**	**Est = -.014, p < .001**	**Est = -.023, p < .001**	39.4%
Fp2 Electrode	**Est = -.006, p = .002**	**Est = -.017, p < .001**	**Est = -.023, p < .001**	26.0%

Note. Average causal mediation effects (ACME) denote the mediation effect, average direct effect (ADE) is the effect of the group after controlling for the mediator, and total effects indicate the effects of group without the mediator. Bold text denotes analyses that survive Bonferroni correction.

Table S3. Skull thickness mediations.

	Average Causal Mediation Effect	Average Direct Effect	Total Effect	Proportion Mediated
		Fp1-Fp2 Left dlPFC		
Left dlPFC	**Est = -.002, p = .002**	Est = -.005, p = .016	**Est = -.007, p < .001**	32.9%
Fp1 Electrode	**Est = -.002, p = .004**	Est = -.005, p = .010	**Est = -.007, p < .001**	28.0%
Fp2 Electrode	**Est = -.003, p = .002**	Est = -.005, p = .034	**Est = -.007, p = .002**	36.4%
		Fp1-Fp2 Right dlPFC		
Right dlPFC	Est = -.002, p = .008	**Est = -.008, p < .001**	**Est = -.010, p < .001**	18.1%
Fp1 Electrode	Est = -.002, p = .020	**Est = -.008, p = .002**	**Est = -.010, p < .001**	20.4%
Fp2 Electrode	**Est = -.003, p < .001**	**Est = -. 007, p = .002**	**Est = -.010, p < .001**	29.0%
		F3-Fp2 Left dlPFC		
Left dlPFC	Est = -.012, p = .006	**Est = -.019, p = .002**	**Est = -.031, p < .001**	39.1%
F3 Electrode	**Est = -.012, p < .001**	**Est = -.019, p = .004**	**Est = -.031, p < .001**	39.5%
Fp2 Electrode	**Est = -.009, p = .002**	**Est = -.022, p = .002**	**Est = -.030, p < .001**	28.5%
		F3-Fp2 Right dlPFC		
Right dlPFC	Est = -.005, p = .006	**Est = -.017, p < .001**	**Est = -.023, p < .001**	23.2%
F3 Electrode	**Est = -.006, p = .002**	**Est = -.017, p < .001**	**Est = -.023, p < .001**	26.6%
Fp2 Electrode	**Est = -.006, p = .002**	**Est = -.017, p < .001**	**Est = -.023, p < .001**	27.5%

Note. Average causal mediation effects (ACME) denote the mediation effect, average direct effect (ADE) is the effect of the group after controlling for the mediator, and total effects indicate the effects of group without the mediator. Bold text denotes analyses that survive Bonferroni correction.

Table S4. CSF thickness mediations.

	Average Causal Mediation Effect	Average Direct Effect	Total Effect	Proportion Mediated
		Fp1-Fp2 Left dlPFC		
Left dlPFC	Est = -.001, p = .352	**Est = -.006, p = .002**	**Est = -.007, p < .001**	10.8%
Fp1 Electrode	Est = -.002, p = .006	Est = -.005, p = .010	**Est = -.007, p < .001**	27.2%
Fp2 Electrode	Est = -.001, p = .012	**Est = -.006, p = .004**	**Est = -.007, p < .001**	17.9%
		Fp1-Fp2 Right dlPFC		
Right dlPFC	Est = -.001, p = .072	Est = -.008, p < .001	**Est = -.010, p < .001**	12.3%
Fp1 Electrode	Est = -.002, p = .026	Est = -.008, p < .001	**Est = -.010, p < .001**	20.9%
Fp2 Electrode	Est = -.002, p = .110	Est = -.008, p < .001	**Est = -.010, p < .001**	15.7%
		F3-Fp2 Left dlPFC		
Left dlPFC	Est = -.003, p = .370	**Est = -.027, p < .001**	**Est = -.030, p < .001**	11.1%
F3 Electrode	Est = -.002, p = .400	**Est = -.028 p < .001**	**Est = -.030, p < .001**	6.6%
Fp2 Electrode	Est = -.005, p = .120	**Est = -.025, p < .001**	**Est = -.030, p < .001**	15.7%
		F3-Fp2 Right dlPFC		
Right dlPFC	Est = -.003, p = .054	**Est = -.019, p < .001**	**Est = -.023, p < .001**	14.7%
F3 Electrode	Est = -.001, p = .420	**Est = -.021, p < .001**	**Est = -.023, p < .001**	4.3%
Fp2 Electrode	Est = -.003, p = .120	**Est = -.019, p < .001**	**Est = -.023, p < .001**	14.9%

Note. Average causal mediation effects (ACME) denote the mediation effect, average direct effect (ADE) is the effect of the group after controlling for the mediator, and total effects indicate the effects of group without the mediator. Asterisk denotes analyses that survive Bonferroni correction.

Table S5. BMI mediation of scalp, skull, and CSF thicknesses.

	Average Causal Mediation Effect	Average Direct Effect	Total Effect	Proportion Mediated
Scalp				
Left dlPFC	**Est = .513, p < .001**	Est = .290, p = .07	**Est = .803, p < .001**	63.9%
Right dlPFC	**Est = .509, p < .001**	Est = .435, p = .012	**Est = .945, p < .001**	53.9%
Fp1 Electrode	**Est = .376, p < .001**	Est = .164, p = .29	**Est = .540, p < .001**	69.7%
Fp2 Electrode	**Est = .389, p < .001**	Est = .216, p = .150	**Est = .604, p < .001**	64.3%
F3 Electrode	**Est = .475, p < .001**	Est = .141, p = .270	**Est = .616, p < .001**	77.1%
Skull				
Left dlPFC	**Est = .432, p < .001**	Est = .242, p = .32	**Est = .674, p < .001**	64.0%
Right dlPFC	**Est = .404, p < .001**	Est = .213, p = .336	**Est = .617, p = .002**	65.5%
Fp1 Electrode	Est = .362, p = .014	Est = .468, p = .116	**Est = .830, p = .002**	43.6%
Fp2 Electrode	Est = .361, p = .008	Est = .651, p = .048	**Est = 1.01, p = .002**	35.7%
F3 Electrode	**Est = .516, p < .001**	Est = .399, p = .160	**Est = .915, p = .002**	56.4%
CSF				
Fp1 Electrode	Est = .176, p = .004	Est = .189, p = .214	Est = .365, p = .006	48.2%

Note. Average causal mediation effects (ACME) denote the mediation effect, average direct effect (ADE) is the effect of the group after controlling for the mediator, and total effects indicate the effects of group without the mediator. Bold text denotes analyses that survive Bonferroni correction.

Table S6. Comparison of electric field strength (V/m) with 10 mm vs. 20 mm regions-of-interest.

		10 mm	20 mm	Statistic
Control				
	Fp1-left	.091 (.012)	.093 (.012)	**V = 126, p < .001, d = .180**
	Fp1-right	.093 (.014)	.095 (.013)	**V = 107, p < .001, d = .187**
	F3-left	.225 (.042)	.232 (.038)	**V = 123, p < .001, d = .154**
	F3-right	.198 (.026)	.202 (.026)	**V = 218, p = .002, d = .138**
Psychosis				
	Fp1-left	.084 (.014)	.086 (.014)	**V = 1298, p < .001, d = .136**
	Fp1-right	.083 (.014)	.086 (.014)	**V = 864, p < .001, d = .171**
	F3-left	.193 (.046)	.200 (.046)	**V = 550, p < .001, d = .157**
	F3-right	.174 (.029)	.177 (.029)	**V = 1835, p < .001, d = .106**
Relative				
	Fp1-left	.086 (.013)	.088 (.014)	**V = 234, p < .001, d = .195**
	Fp1-right	.086 (.014)	.088 (.014)	**V = 257, p < .001, d = .159**
	F3-left	.207 (.042)	.217 (.043)	**V = 79, p < .001, d = .24**
	F3-right	.188 (.029)	.191 (.029)	**V = 585, p < .001, d = .106**
Control vs. Psychosis				
	Fp1-left	**Estimate = -.007, SE = .002, t = 3.13, p = .002, f^2 = .050**	**Estimate = -.007, SE = .002, t = 3.22, p = .002, f^2 = .053**	
	Fp1-right	**Estimate = -.009, SE = .002, t = 4.26, p < .001, f^2 = .097**	**Estimate = -.009, SE = .002, t = 4.30, p < .001, f^2 = .099**	

	F3-left	**Estimate = -.031, SE = .008, t = 4.06, p < .001, f^2 = .088**	**Estimate = -.031, SE = .007, t = 4.12, p < .001, f^2 = .091**
	F3-right	**Estimate = -.023, SE = .005, t = 4.93, p < .001, f^2 = .132**	**Estimate = -.023, SE = .012, t = 5.06, p < .001, f^2 = .140**
Control vs. Relative			
	Fp1-left	Estimate = -.003, SE = .002, t = 1.10, p = .272, f^2 = .002	Estimate = -.002, SE = .002, t = .905, p = .367, f^2 = .002
	Fp1-right	Estimate = -.004, SE = .003, t = 1.38, p = .171, f^2 = .008	Estimate = -.004, SE = .003, t = 1.49, p = .140, f^2 = .011
	F3-left	Estimate = -.009, SE = .007, t = 1.27, p = .208, f^2 = .005	Estimate = -.007, SE = .007, t = .973, p = .333, f^2 = .001
	F3-right	Estimate = -.008, SE = .005, t = 1.50, p = .136, f^2 = .011	Estimate = -.008, SE = .005, t = 1.57, p = .119, f^2 = .013

Note. We used Wilcoxon signed-rank tests for the paired tests comparing 10 mm vs. 20 mm.

Table S7. Mediation analyses of scalp-to-cortex thickness & body mass index on electric field strength using 20 mm regions-of-interest.

	Average Causal Mediation Effect	Average Direct Effect	Total Effect	Proportion Mediated
		Fp1-Fp2 Left dlPFC		
Left dlPFC	**Est = -.004, p < .001**	Est = -.003, p = .146	**Est = -.007, p = .004**	55.4%
Fp1 Electrode	**Est = -.006, p < .001**	Est = -.002, p = .348	**Est = -.007, p = .002**	74.5%
Fp2 Electrode	**Est = -.005, p < .001**	Est = -.002, p = .32	**Est = -.007, p < .001**	72.5%
BMI	**Est = -.004, p < .001**	Est = -.004, p = .11	**Est = -.007, p < .001**	50.9%
		Fp1-Fp2 Right dlPFC		
Right dlPFC	**Est = -.004, p < .001**	Est = -.006, p = .012	**Est = -.010, p < .001**	41.8%
Fp1 Electrode	**Est = -.005, p < .001**	Est = -.004, p = .022	**Est = -.010, p < .001**	54.0%
Fp2 Electrode	**Est = -.006, p < .001**	Est = -.004, p = .046	**Est = -.010, p < .001**	56.0%
BMI	**Est = - .004, p < .001**	**Est = - .006, p = .004**	**Est = -.010, p < .001**	38.2%
		F3-Fp2 Left dlPFC		
Left dlPFC	**Est = -.024, p < .001**	Est = -.006, p = .17	**Est = -.031, p < .001**	79.7%
F3 Electrode	**Est = -.024, p < .001**	Est = -.006, p = .19	**Est = -.030, p < .001**	78.7%
Fp2 Electrode	**Est = -.018, p < .001**	Est = -.012, p = .036	**Est = -.031, p < .001**	60.3%
BMI	**Est = -.021, p <.001**	Est = -.009, p = .14	**Est = -.031, p < .001**	68.8%
		F3-Fp2 Right dlPFC		
Right dlPFC	**Est = -.013, p < .001**	Est = -.011, p = .006	**Est = -.023, p < .001**	54.9%
F3 Electrode	**Est = -.013,**	**Est = -.010,**	**Est = -.023,**	55.6%

	**p < .001**	**p < .001**	**p < .001**	
Fp2 Electrode	**Est = -.013, p < .001**	**Est = -.010, p = .004**	**Est = -.023, p < .001**	55.6%
BMI	**Est = - .013, p < .001**	Est = -.011, p = .008	**Est = -.023, p < .001**	53.6%

Note. Average causal mediation effects (ACME) denote the mediation effect, average direct effect (ADE) is the effect of the group after controlling for the mediator, and total effects indicate the effects of group without the mediator. Bold text denotes analyses that survive Bonferroni correction.
